# Adherence to proper blood pressure measurements among interns at the university of Gondar specialized referral hospital

**DOI:** 10.3389/fcvm.2025.1436256

**Published:** 2025-03-12

**Authors:** Mihret Getnet, Amare Belete Getahun, Desalegn Anmut Bitew, Ayechew Adera Getu

**Affiliations:** ^1^ Department of Human Physiology, School of Medicine, College of Medicine and Health Sciences, University of Gondar, Gondar, Ethiopia; ^2^ Department of Epidemiology and Biostatistics, Institute of Public Health, College of Medicine and Health Sciences, University of Gondar, Gondar, Ethiopia; ^3^ Department of Anesthesia, College of Medicine and Health Science, University of Gondar, Gondar, Ethiopia; ^4^ Department of Reproductive Health, Institute of Public Health, College of Medicine and Health Science, University of Gondar, Gondar, Ethiopia

**Keywords:** adherence, interns, Ethiopia, blood presssure, measurement

## Abstract

**Introduction:**

Blood pressure is a lateral force exerted on the wall of arteries and is critical for the normal distribution of blood containing nutrients and oxygen to metabolic tissues. It is one of the vital signs often measured by interns, nurses, and physicians at doctor’s offices, at bedside, and possibly at home. Accurate blood pressure measurement is essential for proper diagnosis and management of patients, especially those with hypertension. The aim of this cross-sectional survey study is to assess the practice of measuring blood pressure by interns.

**Methods:**

This study was conducted in the form of a survey administered through face-to-face interviews. All the interns at the Univeristy of Gondar Specialized Referral Hospital were approached. The survey included questions about devices used, patient's information, and blood pressure measurement techniques. Epi-Data version 3.1 was used for data entry and exported to STATA 17 for data management and analysis. The Chi-square test was checked to assess the eligibility of variables for logistic regression. Finally, in the multivariable binary logistic regression analysis, variables with *P*-value < 0.05 were considered to be statistically significantly associated. The Adjusted Odds Ratio (AOR) with 95% CI was reported to declare the statistical significance and strength of association between blood pressure measurement and independent variables.

**Result:**

The magnitude of appropriate measurement of blood pressure among interns was 10.1 (95% CI: 7.19, 13.9). A total of 318 interns participated in the current study. Of these study participants, 65.4% (208) were males. A increase in participants age (AOR: 1.48, 95% CI: 1.09, 2.01), being male interns (AOR: 5.51, 95% CI: 1.51, 8.97), and having patients who were familiar with the procedure (AOR: 2.95, 95% CI: 1.19, 7.03) were factors significantly associated with appropriate adherence to blood pressure measurement.

**Conclusion and recommendation:**

Only 10% of six-year medical students (Interns) were successful in appropriately assessing blood pressure. Age, being male, and patient understanding were factors significantly associated to the adherence of blood pressure measurement. Considering the frequency of BP measurement and the impact of hypertension on morbidity and mortality, efforts are needed to maximize the quality of BP measurement by health professionals. This process should begin early during training and be consistent throughout their clinical practice, supplemented by ongoing education.

## Introduction

### Statement of the problem

Despite improvements in therapeutic interventions, it is anticipated that cardiovascular disease (CVD) will continue to be the world's leading cause of morbidity and mortality (1). More deaths and illnesses globally are caused by hypertension than by any other biological risk factor (2). Blood pressure measurement is a basic clinical procedure. However, numerous studies have shown that many errors are made due to the incorrect performance of procedures (3).

Although one of the most inaccurate metrics, blood pressure measurement is still the most crucial in clinical medicine (4). Readings obtained using the auscultatory method by a trained healthcare professional have always been considered the gold standard for clinical blood pressure measurement (5). An important factor in the overall burden of adult diseases in any population is the impact of untreated or mistreated hypertension on the health of patients (6). However, if the illness is identified early and treated reasonably, the consequences of poorly managed hypertension may be avoided (7). However, research conducted globally has shown that there is a lack of understanding of the techniques and skills required to determine blood pressure (8–10). The considerable deficit of ability to measure blood pressure and the lack of understanding of the indirect arterial method of blood pressure measurement was demonstrated in a study to ascertain whether there was a knowledge deficit among healthcare workers regarding BP and its measurement (11). Similar to this, a study was carried out in Ontario to see if a training program for blood pressure measurement would improve knowledge and skills (12).

Improper cuff size, inaccurate patient positioning, rapid cuff deflation, terminal digit preference or bias, monitor not being at eye level, subjects' rest period prior to BP measurement, and the documentation of any abnormal BP patterns were among the common knowledge gaps associated with measurement error (13). Ambulatory care settings have also been shown to rarely use suggested approaches to test blood pressure for the diagnosis of hypertension (14, 15). Researchers discovered that first-year family practice residents (doctors in their first year of practice) and interns (family practitioners who have just finished a six-year degree) show substantial weaknesses in their knowledge and usage of the suggested ways to monitor blood pressure (16, 17). The results related to shortcomings in the training of BP measuring techniques in Canadian medical schools, which could lead to erroneous diagnosis and management of high BP (18). In Canada (19), the American Heart Association's (AHA's) guidelines for BP measurement methodologies were followed only 42% of the time. A number of the previous studies were carried out abroad and adhered to the AHA's guidelines. None of the studies addressed the South African context or the European Society of Hypertension (ESH) recommendations, which are typically implemented in the majority of South African healthcare facilities. The AHA and ESH guidelines slightly differ from one another. The AHA advises taking a minimum of two blood pressure measures with a one-minute interval between them while recording the average of the readings (AHA, 2019). The ESH (2018) highly recommends repeated readings in suspected hypertensive patients at 1 and 3 min (down from 5 min) after standing. In South African healthcare settings, the primary healthcare approach is taken and the crucial role that nursing staff play in the management and treatment of hypertension is acknowledged (20). Therefore, the aim of our study is to assess the adherence of interns (six-year degree) to the proper measurements of blood pressure.

Furthermore, we consider the following research sub-hypotheses:

I. H_0_: there is no significant association between knowledge of guidelines and adherence to blood pressure measurement practices among interns
  H_A_: there is a significant association between knowledge of guidelines and adherence to blood pressure measurement practices among interns
II. H_0_: there is no significant difference in adherence to proper blood pressure measurement practices among interns from different clinical rotations
  H_A_: there is a significant difference in adherence to proper blood pressure measurement practices among interns from different clinical rotations

## Methods

### Participants

A list of all rotating interns was obtained from the registrar office of the University of Gondar. Of the 12 interns who had graduated in the previous year, five were excluded due to prior knowledge of the objectives of the study. A further ten subjects had out-of-town postings and were unable to participate. An appointment was made with individual interns in an out-patient setting. Interns were questioned to determine when they received instruction on blood pressure measurement. A standard introduction of the study was given to all the participating intern-doctors requested to complete the questionnaire. Participants were asked to concentrate on the assessment of blood pressure rather than spend time on history and general examination. The study sites were each equipped with an examination table, mercury manometer, and medium and large adult size cuffs. Only factors documented to have major effects on blood pressure readings as well as proper means of approaching were assessed in this study. Whether or not the intern allowed the patient to rest for an adequate time and measured systolic blood pressure by palpation was noted. Once the cuff size had been selected, patient position and site of measurement were recorded. Training on BP measurement for intern doctors was given early in their journey of medical education, with full practical activity in their basic science laboratory session. The questionnaire tested knowledge on the current recommendations with regard to length of rest prior to blood pressure measurement, effect of arm support, effect of arm position relative to the heart level, appropriate cuff deflation rate, importance of a blood pressure measurement by palpation, appropriate patient positions for blood pressure measurements, and importance of bilateral blood pressure readings.

During data analysis, interns' were credited with correct technique only if they followed the recommended techniques consistently throughout all blood pressure measurement and readings. The results of study participants were compared using the *x*^2^-test to check associations with appropriate adherence of BP measurement and its eligibility to logistic regression. Finally, in the multivariable binary logistic regression analysis, variables with *P*-value < 0.05 were considered to have statistically significant association. The Adjusted Odds Ratio (AOR) with 95% CI was reported to declare the statistical significance and strength of association between adherence of blood pressure measurement and independent variables.

## Questionnaire survey

In June and July 2022, a self-administered questionnaire was administered and completed by each participant. The questionnaire was filled after providing written informed consent; it addresses the following content: sociodemographic factors and measurement-related issues (type of device used, repetition of measurement twice or more to obtain the average BP, mean resting time before measurement, approach to patients wearing thin-sleeved shirts, and approach to patients wearing thick clothing).

We calculated the adherence ratio as the percentage of facilities that made the appropriate choices that adhered to BP measurement guidelines (21). Appropriate choices for each question were based on the following criteria: (i) Upper arm devices were used, (ii) Measurement was performed twice or more per sitting to determine the average BP at least 80% of the time, (iii) Proper cuff size was used, (iv) History of food/caffeine intake/smoking was asked, (v) Functionality of Bp-cuff before measurement was checked, (vi) Patient position while measuring, (vii) Which arm was used for measuring Bp, and (viii) Arm was held at heart level. Upper arm devices included sphygmomanometers, semi-automated (manual compression and automatic measurement) upper arm oscillometric devices, and automated upper arm oscillometric devices. Interns that selected one of these upper arm devices were considered to be adherent to the guideline. For the number of measurements performed to obtain average BP, we adopted “measuring twice or more at least 80% of the time” as the standard. Assessment of clothing status was restricted to facilities that used upper arm devices, under the assumption that clothing had less impact when other devices, such as wrist-type devices, were used. The study participants were asked to answer demographic and educational questions, and their ability to measure blood pressure accurately using BP measurement guidelines was assessed in the structured questionnaire. Approval by our college’s institutional review board was obtained before conducting this research. A convenience sample was taken from 6th year practiced medical students at the University of Gondar College of Medicine and health science campus. We independently processed the information collected from the questionnaire. The data was entered to EpiData 3.1 and exported to STATA 17 for further analysis, and finally association was assessed using multivariable logistic regression. Variance inflation factor (VIF) was done to crosscheck the interaction between the independent variables (VIF = 3.23), which is less than 10, and the corresponding model fitness was done using Hosmer and Lemeshow goodness of fit test (*p*-value = 0.4695). This indicates the model is well fitted *p*-value > 0.05 and no multicollinearity effect was observed between the independent variables.

## Results

A total of 318 interns participated in the study. The age of study participants ranged from 22–30 years, with a mean age of 25.45 ± 1.25. Of the participants, 65.4% (208) were males. Almost 97% (309) of patients agreed to have their blood pressure measured. Only half (50%) of the interns provided information on the procedure to their patient. About 38% (111) of interns asked about food/alcohol/cigarette intake history before measurement. Proper BP cuff size was utilized by only 33% (107) of intern doctors. Nearly 70% (221) of interns took the blood pressure measurement while patients were in a sitting position. Moreover, 81% (258) of interns applied arm support at the heart level while measuring blood pressure (Table 1).

**Table 1:** Descriptive analysis of adherence to blood pressure measurement among interns at the University of Gondar, Northwest Ethiopia.

Variables		mean ± Std.dev	Frequencies	Percent (%)
Age	22–30	25.45 ± 1.25	318	100
Sex	Male		208	65.41
	Female		110	34.59
Department	Internal medicine		80	25.16
	Paediatrics		50	15.72
	Surgery		98	30.82
	Gyn/Obs		90	28.30
Attachment Ward	Emergency		55	17.30
	ICU		9	2.13
	OPD		24	7.55
	Labor ward		31	9.75
	PACU/recovery		47	14.78
	Others		152	47.80
Patient willingness	Yes		309	97.17
	Not usually		8	2.52
	No		1	0.31
Patient orientation about measurement	Yes		162	50.94
	Not usually		134	42.14
	No		22	6.92
Ask about food intake before measurement	Yes	Ignore the effect	10	8.26
		Measure after some time	35	28.93
		After 30 min	76	62.81
	Not usually		162	50.94
	No		35	11.01
Type of device you used	Manual sphygmomanometer		109	34.28
	Portable Electronic device		124	38.99
	Automated office BP machine		85	26.73
Proper size of BP cuff	Yes		107	33.65
	Not usually		160	50.31
	No		51	16.04
Have you checked functionality	Yes		189	59.43
	Not usually		100	31.45
	No		29	9.12
How many times did you measure	Once		118	37.11
	Two and above		200	62.89
Patient position during measurement	Supine		82	25.79
	Sitting		221	69.50
	Lateral		13	4.09
	Standing		2	0.63
Preferred arm	Left		115	36.16
	Right		110	34.59
	Either left or right		93	29.25
Reason for preference	Measurement guideline		153	66.81
	Patient preference		26	11.35
	Your personal convenience		50	21.83
Arm support at the level of the heart	Yes		258	81.13
	No		60	18.87

### Magnitude of appropriate adherence to blood pressure measurement among interns

**Figure 1:**
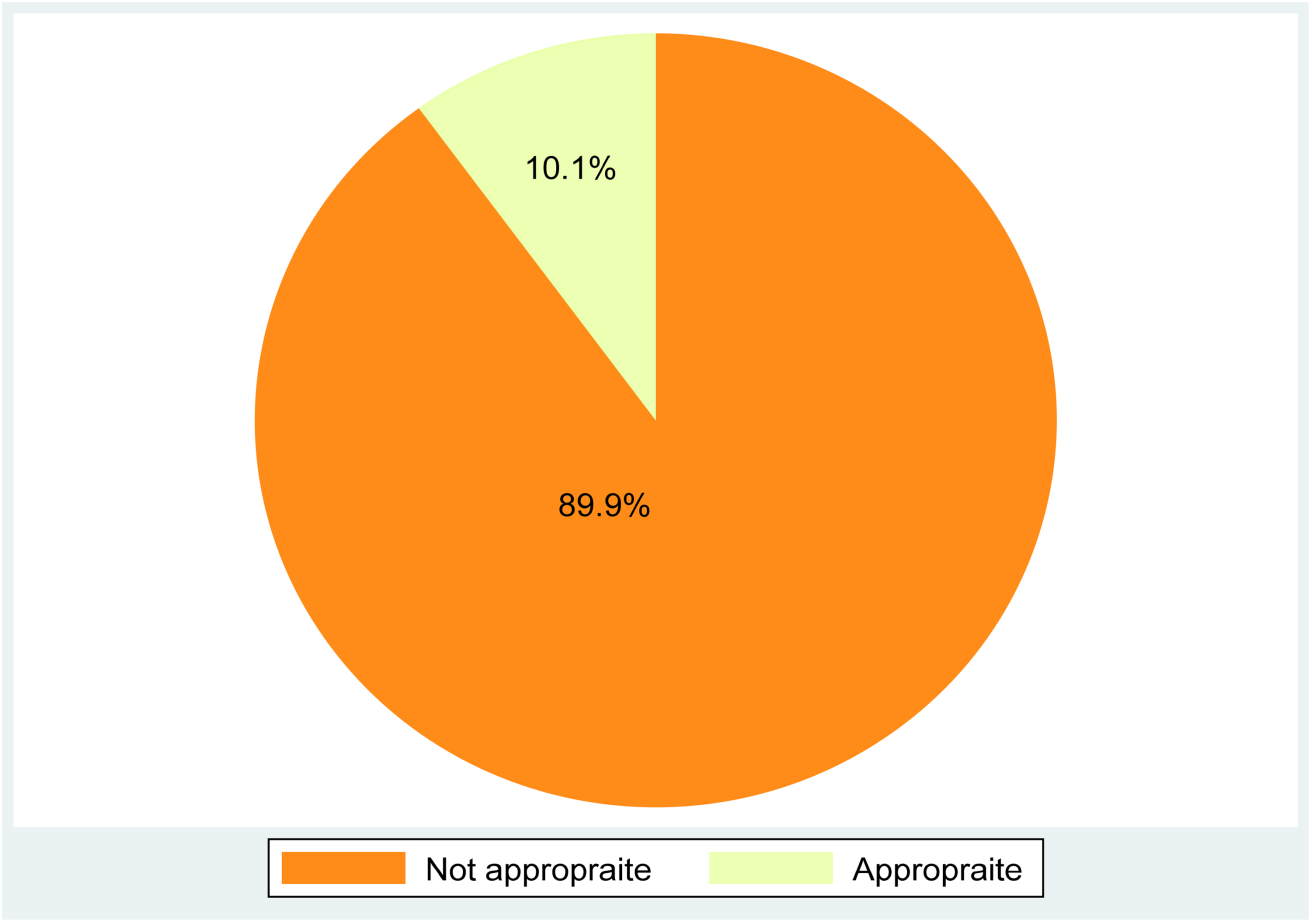
Pie chart showing the magnitude of appropriate adherence to blood pressure measurement guidelines among interns at the University of Gondar, Northwest Ethiopia.

Appropriate blood pressure measurement is a routine activity in medical education. Having a good approach for measuring blood pressure and consistent monitoring is essential for reducing cardiovascular complications among patients. In this study, the prevalence of appropriate adherence to blood pressure measurement among intern doctors was 10.1% (95% CI: 7.19, 13.9) (Figure 1).

### Associated factors of adherence to blood pressure measurement among interns in binary and multivariable logistic regression analyses

Six variables (age, sex, major department, attachment ward, patient willingness, and patient understanding) that were associated with appropriate adherence to blood pressure measurement in bivariable analysis (*p* < 0.2) were entered into a multivariable logistic regression model. In the multivariable logistic regression, three variables were excluded. Age, being male, and patient understanding were predictor variables that had a significant association with appropriate measurement of blood pressure. Indeed, an increase in the participant’s age by one year meant 1.48 times higher odds of appropriate adherence to blood pressure measurement (AOR: 1.48, 95% CI: 1.09, 2.01). The odds of appropriate adherence to blood pressure measurement among male interns was 5.51 times (AOR: 5.51, 95% CI: 1.51, 8.97) higher than that of female intern doctors. The odds of having appropriate adherence to blood pressure measurement among those interns who had given information about the procedure to their patients was 2.95 times (AOR: 2.95, 95% CI: 1.19, 7.03) higher than participants who did not provide information (Table 2).

**Table 2:** Associated factors of adherence to blood pressure measurement guidelines among interns using binary and multivariable logistic regression analyses, University of Gondar, Northwest Ethiopia, *n* = 318.

Variables		Appropriate Adherence to BP		COR 95%% CI	AOR 95% CI
		Yes (%)	No (%)		
Age		32 (10.06)	286 (89.94)	1.63 (1.24, 2.15)	1.48 (1.09, 2.01)**
Sex	Male	29 (13.94)	179 (86.06)	5.78 (1.72, 19.42)	5.51 (1.51, 8.97)**
	Female	3 (2.7)	107 (97.3)	1	1
Department	Internal medicine	9 (11.25)	71 (88.75)	0.93 (0.31, 2.79)	0.52 (0.22, 2.85)
	Paediatrics	6 (12)	44 (88)	1	1
	Surgery	14 (14.29)	84 (85.71)	1.22 (0.44, 3.40)	1.44 (0.40 5.14)
	Gyn/Obs	3 (3.33)	87 (96.67)	0.25 (0.06, 1.06)	0.24 (0.05, 1.13)
Ward attachment	Emergency	8 (14.55)	47 (84.45)	1	1
	ICU/labor	3 (7.5)	37 (92.5)	0.48 (0.12, 1.92)	0.59 (0.09, 3.87)
	OPD	4 (16.66)	20 (83.34)	1.18 (0.32, 4.35)	1.08 (0.22, 5.25)
	PACU/recovery	4 (8.51)	43 (91.49)	0.57 (0.15, 1.95)	0.24 (0.05, 1.17)
	Others*	13 (8.55)	139 (91.45)	0.55 (0.21, 1.41)	0.43 (0.14, 1.31)
Patient willingness	Yes	31 (10.03)	278 (89.97)	0.89 (0.21, 1.41)	1.32 (0.10, 16.83)
	No	1 (11.11)	8 (88.89)	1	1
Patient orientation	Yes	23 (14.20)	139 (85.8)	2.70 (1.21, 6.04)	2.95 (1.19, 7.03)**
	No	9(5.77)	147 (94.23)	1	1

* Others = wards psychiatry, Ophthalmology, ENT (Ear, Nose, and Throat), Dentistry, Dermatology, Radiology.

** *p*-value < 0.05, AOR, adjusted odds ratio; COR, crude odds ratio.

## Discussion

Adherence to proper blood pressure measurement is crucial in the management of patients with hypertension and to diagnose individuals accurately in order to reduce risk factors related to cardiovascular disease. Medical students and clinicians should practice strict blood pressure measurement to monitor the progress of disease during patient follow-up.

In our survey we tried to assess blood measurement techniques, the type of instrument used, arm support at the level of the heart, BP cuff size, patient orientation and position while being measured, repetition of measurement for confirmation, preferred arm, and whether a history of food/alcohol/cigarette consumption were taken. These criteria were adopted from previous literature and in line with appropriate blood pressure measurement (22, 23).

Even though most of the patients visiting the hospital volunteer to have their blood pressure measured, only 50.9% of interns informed patients before measurement. But patients should be given clear information before performing BP measurement to consider patient preferences, values, and clinical factors (24). Informed initiation of BP measurement also allows patients to take preventive action (25). This indicates the current challenge of hypertension management is due to a information gap from the side of clinicians (26) and unimproved patient adherence.

In this study, about 62.8% of interns took BP measurement after resting patients for 30 min, while 29% and 8% took the measurement before 30 min rest and ignored the effect of ingested food/alcohol/cigarettes, respectively. Taking adequate rest before BP measurement is supported by studies done in China (27) and the USA (28). The possible reason may be in conditions of severe illness that needs immediate attention and the BP measurement will done without having stable environment. Errors in blood pressure measurement can result in overestimation, leading to unnecessary treatment and potential exposure to the adverse effects of medication (29).

Nearly 70% of intern doctors took BP measurement with the patient in a sitting position. Even though sitting position appears to be appropriate for BP measurement (30), 30% of interns reported using either lateral, supine, or standing positions. Because blood pressure directly affects the heart, these positions as BP measurement techniques may induce inaccuracies compared to the standard position (31). Additionally, only 66% of interns used the preferred arm for BP measurement per the guidelines, while 34% of interns followed patient preference and their own convenience in selecting either arm for blood pressure measurement. Interns who practiced patient preference or their own judgment to use the preferred arm for BP measurement may result in subjective assessment, which is not in line with standard guidelines.

The current study revealed that appropriate adherence to blood pressure measurement among interns was 10.1% (95% CI: 7.19, 13.9). This finding was similar to a study done in the University of Belgrade, Serbia (32), and to one conducted in Spain among medical and nursing students (33).One possible reason for the findings may be that the theoretical knowledge of participants was inadequate to perform blood pressure measurement in a standardized manner and prevent introduced errors (34). Another reason is workflow constraints (multitasking or inadequate time), patient characteristics (behaviors), and inadequate equipment access for conducting accurate BP measurement and monitoring (35). However, our findings are lower than that of a study done in Colombia among healthcare workers (36). Even though inadequate knowledge of correct BP measurement procedures is unlikely to improve during specialized postgraduate training or in-service courses, because it is a basic clinical procedure that students are assumed to have learned in Health Science faculties (33), healthcare practitioners in Colombia had longer exposure to clinical practice in major hospitals regarding good adherence to blood pressure measurement. Therefore, performing BP measurements repeatedly in accordance with standard guidelines may improve intern doctors and other clinicians' ability to appropriately measure BP.

Per our findings, age, being male, and giving information before measurement were statistically significant factors associated with appropriate adherence to blood pressure measurement guidelines among interns. A one-year increase in age increases the odds of appropriate adherence to blood pressure measurement by 1.48 among graduating medical students (interns). This finding is supported by a study conducted in Serbia (32) and in Lowa City college of Medicine (37). One reason for the findings may be that, as seniority increases, there is a reciprocal approach to measure blood pressure based on the guidelines among physicians in the United States (37). Health professionals who have worked in the field for a long time will be familiar with patient behaviors' and build a capacity to multitask, ensuring accurate BP measurement.

In this study, being male was significantly associated with appropriate adherence to blood pressure measurement guidelines among six-year graduating medical students. A study done in the Philippines among nursing students showed that being female has a significant association with incorrect adherence to blood pressure measurement (38). This may be due to significant differences in the proportion of sex among study participants and patients’ reactions to female intern medical students.

Moreover, it was investigated that giving information about BP measurement to patients was more likely to result in appropriate adherence to BP measurement guidelines. Similarly, previous studies identified familiarizing patients about the procedure as a positive (35, 39). Giving a brief orientation to patients regarding BP by considering blood pressure measurement guidelines establishes a good relationship between patients and healthcare professionals, which aids the correct and accurate measurement of blood pressure (40). Smoking within 30 min of measurement, having a distended bladder, sitting with both legs are crossed, and talking/listening during measurement can raise blood pressure measurements by various degrees. Therefore, patient understanding about these related factors allows healthcare professionals to record correct blood pressure (41).

## Conclusion

This study indicates that final year graduating medical students (Interns), regardless of their attachment ward or experience, measured blood pressure inadequately, incorrectly, and inaccurately. Even though measurement techniques or guidelines are required to make sure that blood pressure readings are accurate, consistent, and repeatable, correct measurement of BP is affected by a wide range of factors and is challenging to accomplish consistently in busy medical centers. Considering the frequency of BP measurement and the impact of hypertension on morbidity and mortality, efforts are needed to maximize the quality of BP measurement by healthcare professionals. To address these concerns, procedures should be implemented to increase the use of proper technique for blood pressure measurement. This process should begin early during training and be maintained throughout clinical practice, supplemented by ongoing education. A follow-up study with additional instruction on blood pressure measurement would also be beneficial for medical students before and after graduation.

## Data Availability

The original contributions presented in the study are included in the article/Supplementary Material, further inquiries can be directed to the corresponding author.
