# Supplementary material for: Adherence to proper blood pressure measurements among interns at the university of Gondar specialized referral hospital
Source: Front Cardiovasc Med. 2025 Mar 12;12:1436256. doi: 10.3389/fcvm.2025.1436256 (PMC11937000; doi:10.3389/fcvm.2025.1436256)
Supplement: Supplementary file 1 [file Datasheet1.pdf]

**Survey Questions**:

1. Demographic characteristics
   1.1. Age
   1.2. Sex
2. Department/ward/ attachment during time of data collection ……………
3. Patient information
   3.1. Patient preference to take blood pressure(e.g chronic cases, emergency etc)
   3.2. Do you take history of food/caffeine intake /smoking before measuring
4. Devices used
   4.1. Manual sphygmomanometer
   4.2. Portable Electronic device
   4.3. Automated office BP machines
   4.4. Do you regularly check/calibrate the device before use?
   4.5. How many times you usually measured in a single encounter?
      a. Once
      b. Two times and above
   4.6. What position you usually used?
      a. Supine
      b. Sitting
      c. Lateral
      d. Standing
   4.7. On which arm you usually measure?
      a. Left
      b. Right
   4.8. What was the reason for arm preference?
      a. Measurement guideline/recommendation
      b. Patient's preference
      c. Your personal convenience
   4.9. Do you support the arm to be fixed at the level of the heart?
      a. Yes
      b. Not always
      c. No
